# Supplementary material for: Strong coupling and induced transparency at room temperature with single quantum dots and gap plasmons
Source: Nat Commun. 2018 Oct 1;9:4012. doi: 10.1038/s41467-018-06450-4 (PMC6167320; doi:10.1038/s41467-018-06450-4)
Supplement: Supplementary file 1 — Supplementary Information [file 41467_2018_6450_MOESM1_ESM.pdf]

Supplementary Information for Strong coupling and induced transparency at room temperature with single quantum dots and gap plasmons

Haixu Leng,[1] Brian Szychowski,[2] Marie-Christine Daniel,[2] and Matthew Pelton[1,]*

[1]Department of Physics, UMBC (University of Maryland, Baltimore County), Baltimore, MD 21250, U.S.A.
[2]Department of Chemistry & Biochemistry, UMBC (University of Maryland, Baltimore County), Baltimore, MD, 21250, U.S.A.
*Corresponding author. Email: mpelton@umbc.edu

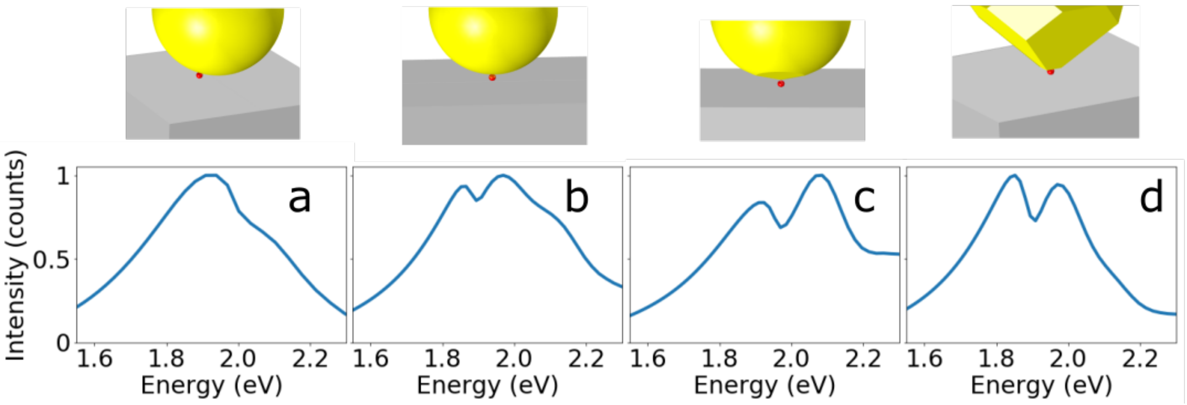

Supplementary Figure 1: Finite-element simulations of coupled quantum-dot / gap-plasmon systems in different coupling regimes. a Quantum dot located next to the gap between a spherical gold nanoparticle and a silver film. **b** Quantum dot in the gap between a spherical gold nanoparticle and a silver film. **c** Quantum dot in the gap between the edge of a faceted gold nanoparticle and a silver film. The facets are formed by truncating a spherical particle with planes. **d** Quantum dot in the gap between the apex of a faceted gold nanoparticle (approximated as a polygon) and a silver film. The top panels show the simulated structures; gold nanoparticles are in yellow, quantum dots are in red, and silver films are in grey. The bottom panels show the simulated scattering spectra.

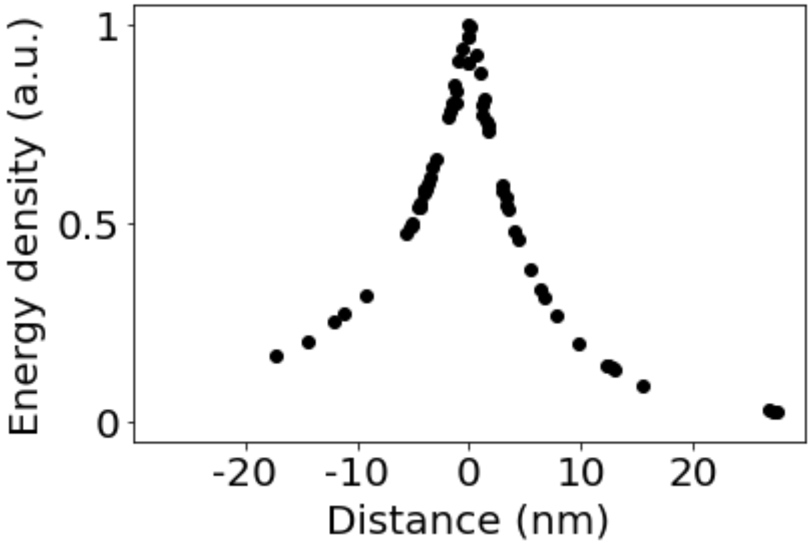

Supplementary Figure 2: Lateral field localization for a gap plasmon. The plot shows the calculated electromagnetic energy density in the gap between a faceted gold nanoparticle and a silver film, as a function of lateral distance (parallel to the surface of the film).

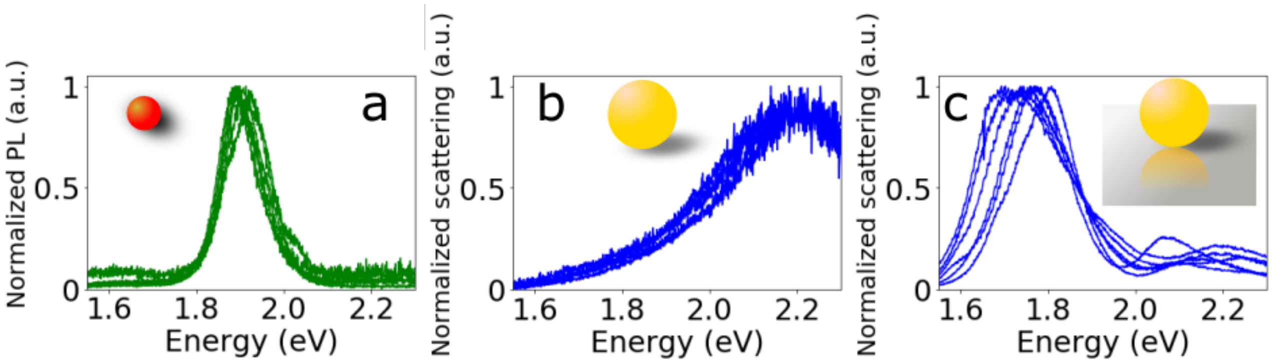

Supplementary Figure 3: Reference spectra for uncoupled particles. a, Photoluminescence from several isolated quantum dots. **b**, Scattering spectra of several isolated quasi-spherical gold nanoparticles. **c**, Scattering spectra of several individual gold nanoparticles on a silver film (without quantum dots).

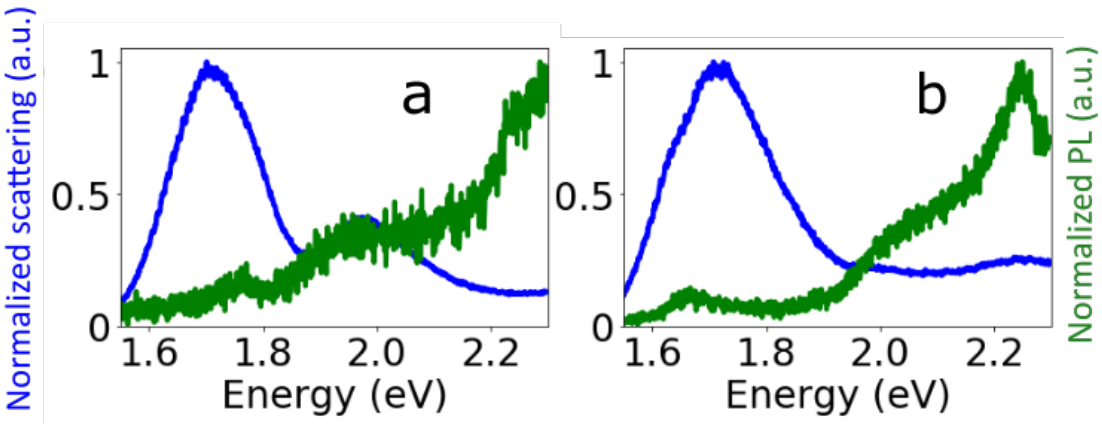

Supplementary Figure 4: Photoluminescence from gap plasmons. Scattering (blue) and photoluminescence (green) spectra from gold nanoparticles on a silver film, without quantum dots. **a** and **b** show representative examples for two different nanoparticles.

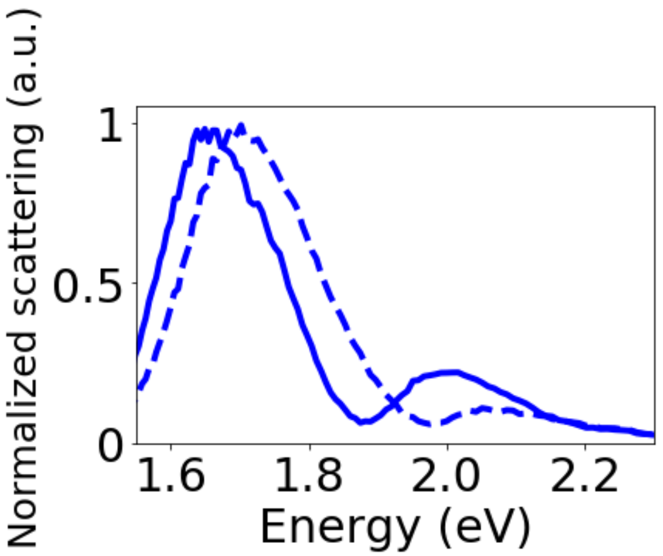

Supplementary Figure 5: Shift of the gap plasmon under intense laser illumination. Scattering spectrum from a gold nanoparticle on a silver film, without quantum dots, before (solid line) and after (dashed line) illumination for 30 seconds with 0.4 mW of 515-nm laser light focused to a 5 µm spot.

Supplementary Table 1: Parameters obtained from fitting of experimental scattering spectra. Quantum dot resonance frequency, ω_{QD}, quantum dot decay rate, γ_{QD}, plasmon resonance frequency, ω_{SP}, plasmon decay rate, γ_{SP}, and coupling strength, *g*. All values are in units of eV.

	ω_{SP}	ω_{QD}	γ_{SP}	γ_{QD}	g
Weak coupling: Figure 3	1.82	1.85	0.20	0.06	0.01
Intermediate coupling: Figure 4a,c	1.93	1.93	0.20	0.06	0.10
Intermediate coupling: Figure 4b,d	2.00	2.03	0.23	0.08	0.11
Strong coupling: Figure 5a,c	1.86	1.88	0.15	0.10	0.23
Strong coupling: Figure 5b,d	1.90	1.91	0.19	0.13	0.16
